# How to Treat Involvement of the Central Nervous System in Hemophagocytic Lymphohistiocytosis?

**DOI:** 10.1007/s11940-017-0439-4

**Published:** 2017-02-02

**Authors:** AnnaCarin Horne, Ronny Wickström, Michael B. Jordan, E. Ann Yeh, Ahmed Naqvi, Jan-Inge Henter, Gritta Janka

**Affiliations:** 1 Department of Women’s and Children’s Health, Karolinska Institute, Division of Pediatrics Karolinska University Hospital, Stockholm, Sweden; 2 Department of Pediatrics, Cincinnati Children’s Hospital Medical Center and the University of Cincinnati College of Medicine, Cincinnati, OH USA; 3 Division of Neurology, Department of Pediatrics, The Hospital for Sick Children, University of Toronto, Toronto, ON Canada; 4 Division of Hematology/Oncology, Department of Pediatrics, The Hospital for Sick Children, University of Toronto, Toronto, ON Canada; 5 Department of Pediatric Hematology and Oncology, University Medical Center Hamburg Eppendorf, Hamburg, Germany

**Keywords:** Hemophagocytic lymphohistiocytosis (HLH), Central nervous system (CNS), Cerebrospinal fluid, Neuroradiology, Treatment, Intrathecal treatment, Overall survival, Long-term effects

## Abstract

Central nervous system (CNS)-hemophagocytic lymphohistiocytosis (HLH) is not a disease in itself, but it is part of a systemic immune response. The vast majority of patients with CNS-HLH also have systemic HLH and a large number of patients with primary and secondary HLH have CNS involvement. Reactivations within the CNS are frequent during the course of HLH treatment and may occur concomitant with or independent of systemic relapses. It is also important to consider primary HLH as an underlying cause of “unknown CNS inflammation” as these patients may present with only CNS disease. To initiate proper treatment, a correct diagnosis must be made. A careful review of the patient’s history and a thorough neurological examination are essential. In addition to the blood tests required to make a diagnosis of HLH, a lumbar puncture with cerebrospinal fluid (CSF) analysis and magnetic resonance imaging (MRI) should always be done in all cases regardless of the presence or absence of neurological signs or symptom. Treatment options for CNS-HLH include, but are not limited to, those commonly used in systemic HLH, including corticosteroids, etoposide, cyclosporine A, alemtuzumab, and ATG. In addition, intrathecal treatment with methotrexate and corticosteroids has become a standard care and is likely to be beneficial. Therapy must be initiated without inappropriate delay to prevent late effects in HLH. An interesting novel approach is an anti-IFN-gamma antibody (NI-0501), which is currently being tested. Hematopoietic stem cell transplantation (HSCT) also represents an important CNS-HLH treatment; patients with primary HLH may benefit from immediate HSCT even if there is active disease at time of transplantation, though care should be taken to monitor CNS inflammation through HSCT and treat if needed. Since CNS-HLH is a condition leading to the most severe late effects of HLH, early expert consultation is recommended.

## Introduction

Hemophagocytic lymphohistiocytosis (HLH) is not a single disease but rather a clinical syndrome of life-threatening hyperinflammation. It may result from genetic defects (primary HLH [1•]) or be acquired with infectious, neoplastic, autoinflammatory, autoimmune, and immunodeficiency etiologies (secondary HLH) [1•]. Primary HLH is rare, with an estimated yearly incidence in Sweden of 0.12–0.15 per 100,000 children [2]. The incidence of primary HLH in adults or that of secondary HLH is not studied.

The term “central nervous system (CNS) disease” is frequently used in the HLH-related literature; but to date, there is no consensus regarding its definition. However, most HLH experts agree that an abnormal CSF and/or MRI of the brain, with or without distinct neurological signs or symptoms, define CNS-HLH. Although differences of opinion still exist on how to define CNS disease, there is agreement that the term refers to infiltration of activated lymphocytes and macrophages into the meninges and brain [3]. CNS disease has been divided into three neuropathological stages: stage I with leptomeningeal inflammation, stage II with perivascular infiltration, and stage III with massive tissue infiltration, blood vessel destruction, and tissue necrosis. This infiltration can induce devastating brain lesions in affected patients and is an important cause of mortality and morbidity in HLH [4••, 5].

In both primary and secondary HLH, CNS involvement is a frequent finding at disease onset [6–9]. In addition, disease reactivation, during or after therapy, occurs frequently in the CNS. Overall, CNS disease has been reported in 30–73% of all HLH patients, either at presentation or during the course of the disease [7, 10, 11••]. As the disease may be difficult to diagnose, a high index of suspicion is required when evaluating patients with systemic HLH. The clinical picture of HLH is similar in primary and secondary cases and is characterized by systemic inflammation, markedly elevated cytokine levels and immune-mediated organ damage [12, 13••, 14]. HLH is currently diagnosed by either (1) a proven genetic mutation or (2) fulfilling five out of eight clinical criteria (fever, splenomegaly, cytopenias of at least two cell lines, hypertriglyceridemia and/or hypofibrinogenemia, hyperferritinemia, abnormally low NK-cell activity, high levels of soluble IL-2 receptor, and pathologic evidence of hemophagocytosis in tissues) [15]. By contrast, the clinical presentation of CNS disease in HLH is highly variable [8, 16]. Occurrence of neurological symptoms is not included as a diagnostic criterion of HLH, but it is important to consider HLH in a child with unexplained neurologic manifestations, especially one with fever, pancytopenia, and hepatosplenomegaly.

As survival in patients with HLH has improved markedly [7, 17], it has become increasingly important to thoroughly evaluate long-term sequelae, the most important of which are neurological. Unfortunately, significant motor and cognitive deficits may occur following HLH [16, 18••]. Early recognition and prompt treatment of CNS disease may prevent irreversible CNS injury and are therefore of greatest importance in order to improve the long-term outcome [5, 16]. Neurological outcomes after treatment are, however, unknown as therapy trials have tended to merely focus on survival.

To recommend the best possible treatment for CNS involvement, we need to understand the pathophysiology of CNS-HLH. It is likely similar to that of systemic HLH, i.e., massive hyper-inflammation leading to destruction of brain tissue. Therefore, it is important to constantly reduce inflammatory HLH activity to prevent CNS injury. In addition, insufficient cytotoxicity in primary HLH may result in reduced elimination of virus infected cells; hence, antiviral therapy should be given when possible. The HLH therapy is based on specific immunotherapy and/or chemotherapy regimens followed by hematopoietic stem cell transplantation (HSCT) in primary HLH [7, 15, 17].

Here, we review the available literature on treatment of CNS involvement in HLH, including mechanisms of action and clinical efficacy. We also present suggestions regarding treatment of CNS-HLH.

## Definition of CNS Disease in HLH

The definition of CNS disease in HLH has not been standardized. Importantly, however, retrospective studies suggest that the presence of CNS disease carries key prognostic significance [19], including higher risk of neurological impairment and future disability as well as higher mortality [16, 19]. This lack of standardized definitions limits knowledge about response of CNS disease to therapy. Comparability across cohorts is equally limited for this reason. Evaluation for possible CNS involvement in HLH rests on information from three specific areas of investigation: (a) the presence of neurological signs/symptoms, (b) neuroimaging abnormalities, and (c) evaluation of CSF. In Table 1, we outline literature describing the relative frequency of specific findings in the three above-mentioned categories.

**Table 1:** Summary of articles (of over 20 children) with data on neurological findings and symptoms, neuroradiology findings, and CSF analyses

Reference	Study type	Cohort n (ages)	Neurological symptoms/findings	Pathological neuroradiology	Pathological CSF *
Arico et al. [20]	Retrospective multicenter	122 (0 month–6 years)	*No data*	*No data*	55/94 (58%)
Haddad et al. [9]	Retrospective single center	34 (1 month–4 years)	25/34 (73%)	10/17 (59%)	29/34 (85%)
Trottestam et al. [7]	Prospective treatment study: HLH-94	249	80/245 (33%)	107/203 (53%)	43/135 (32%)
Hirst et al. [21]	Retrospective single center	23 (3 days–9 years)	7/23 (30%)	*No data*	*No data*
Yang et al. [10]	Prospective single center	92 (2 months–16 years)	12/92 (13%)	36/92 (39%)	15/92 (16%)
Kim et al. [19]	Retrospective single center	50 (10 day–17 years)	19/50 (38%)	12/21 (57%)	13/23 (57%)
Jovanovic et al. [11••]	Retrospective single center	30 (1 month–16 years)	14/30 (46%)	4/9 (44%)	17/30 (56%)
Horne et al. [16]	Prospective treatment study: HLH-94	193 (12 days–14 years)	72/193 (37%)	35/115 (30%)	101/193 (52%)
Henter et al. [4••]	Retrospective multicenter	23 (1month–6years)	15/23 (65%)	*No data*	19/21 (90%)
Deiva et al. [8]	Retrospective single center	46 (0 month–15 years)	29/46 (63%)	31/46 (67%)	23/46 (50%)
Rachmandran et al. [6]	Retrospective single center	43 (50 day–14 years)	12/43 (36%)	*No data*	4/12 (33%)
Koh et al. [22]	Retrospective multicenter	251 (0 year–18 years)	41/233 (18%)	38/106 (36%)	26/77 (34)
Dao et al. [23•]	Prospective, single center	89 (2 months–14 years)	Not specified	9/15 (60%)	74/81 (91%)

### Neurological signs/symptoms

Few studies including prospective neurological evaluations of consecutive HLH patients exist [7, 10, 16, 23•]. The findings of these and a number of retrospective efforts have documented the rate of CNS involvement to be in the range of 18–73% [4••, 6, 8, 9, 11••, 24••]. The larger and more systematic of the studies suggest that about 2/3 of all HLH patients (both primary and secondary) experience neurological manifestations [7, 19]. These numbers rest on evaluation of cases that, for the most part, present with systemic HLH and are noted additionally to have CNS features. However, inflammation of the CNS may be the primary and only clinical presentation of HLH [25–30]. The prospective treatment study, HLH-2004, will provide some answers to these questions. This study documents standardized neurological outcomes and is due to be published in 2016. Preliminary results suggest frequent neurological involvement in HLH. The study is closed but data is not yet published.

Reported neurological symptoms and/or signs are severe, sometimes life-threatening, and may occur early on in the course of the disease. Seizures are the most common sign of neurological dysfunction, as seen in 33–83% of children who are reported to have CNS-HLH [8, 10, 11••, 16, 19, 24••]. Mental status changes, described variably as irritability, disturbance of consciousness, and encephalopathy also occur commonly (31–47%), suggesting that gray matter dysfunction is relatively common in this population. Additionally, meningism is reported in approximately one third of patients with neurological findings in some cohorts. Focal neurological signs, such as hemiparesis, cranial neuropathies, and ataxia, are seen in 10–20% of reported cohorts [8, 10, 11••, 16, 19, 24••]. However, comparison between cohorts is difficult due to inconsistency in the rates of neurological symptoms reported.

The differential diagnosis for CNS-HLH is broad and includes acute disseminated encephalomyelitis (ADEM), acute necrotizing encephalopathy (ANE), CNS vasculitis, multiple sclerosis, encephalitis, CNS manifestations of rheumatologic disease (such as systemic lupus erythematosus), and other genetically mediated CNS inflammatory disorders such as interferonopathies.

### Analysis of CSF

CSF abnormalities are seen in a large proportion of HLH cases with or without neurological symptoms. A lumbar puncture should, therefore, be routinely performed in all children where there is a suspicion of HLH and where no contraindications are present. Analysis of CSF should include standard tests (i.e., cells, protein including fractions, glucose, lactate, and microbiology) and a cytospin with examination for hemophagocytosis.

CSF pleocytosis is seen in 10–47% of HLH patients [7, 10, 11••, 16, 19]. It should be noted, however, that pleocytosis may be a late sign and repeat lumbar punctures may be of value if clinical suspicion remains. Although increased protein levels, as seen in 11–41% of HLH patients [7, 9, 16], are usually only moderately elevated (between 500 and 1000 mg/L, normal range age-dependent 150–400 mg/L), values up to 10,000 mg/L have been reported [10, 31]. Protein levels higher than 2500 mg/L have been associated with stage III abnormalities [4••], but the prognostic value is uncertain as even patients with extremely elevated levels have had a good outcome [31]. High CSF protein levels in an encephalopathic child with unknown diagnosis should raise the suspicion of a neuroinflammatory condition including HLH.

“Abnormal CSF” is defined in many studies as including pleocytosis, increased CSF protein, or both. This classification leads to findings of CSF abnormalities in 16–76% of HLH cases [7, 8, 10, 16]. As a whole, the presence of neurological symptoms and CSF abnormalities of any kind is a negative prognostic marker, e.g., reducing 5-year survival from 67 to 40% [7, 16]. CSF abnormalities may respond rapidly to therapy, with one case series showing resolution of CSF abnormalities in all children within 6 weeks of treatment [10].

Although the pathogenesis of HLH is not fully understood, the clinical symptoms are considered to be mediated by excessive activation of CD8+ T lymphocytes and the release of cytokines such as tumor necrosis factor-α, IL-1β, IL-6, IL-8, and interferon-γ [32–35]. Limited information from cases reports suggests that other neuroinflammatory markers such as neopterin may be useful for diagnosis of CNS disease [36]. Given similarities with other neurological diseases affecting white matter, biomarkers known to be important for these disorders, e.g., CXCL 13 and neurofilament light chain, should also be studied in the future [37]. Since neurological symptoms may be seen in the presence or absence of elevated CSF protein or pleocytosis [12, 15, 16], finding biomarkers with a higher sensitivity and specificity for CNS HLH would be of great importance.

Hemophagocytosis, which is described to be present in 91% of brain biopsies, mostly located in the meninges [4••] and in 92% of bone marrow samples, was less commonly seen in the CSF (39%) of pediatric cases [7]. Whether the degree of hemophagocytosis in the CSF correlates to the duration and severity of disease, as demonstrated in brain tissue [4••], is not known.

### Neuroimaging

**Fig. 1:**
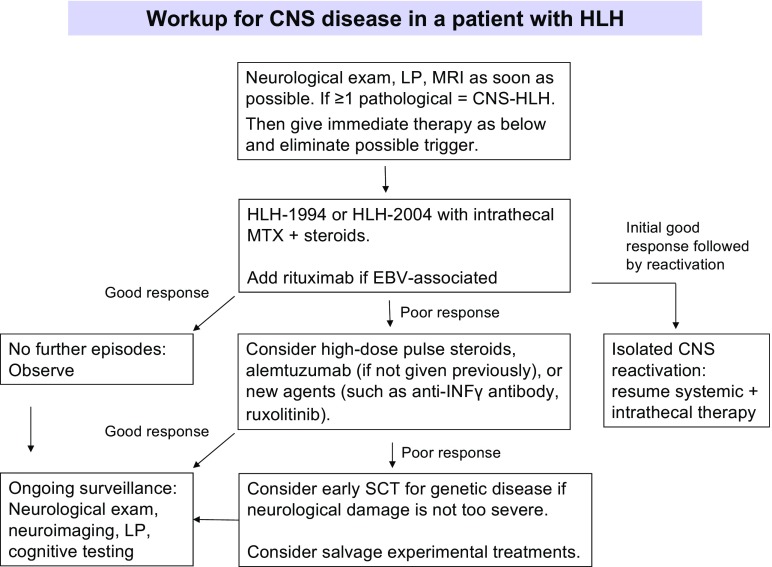
Suggested workup for CNS disease in a patient with HLH.

**Fig. 2:**
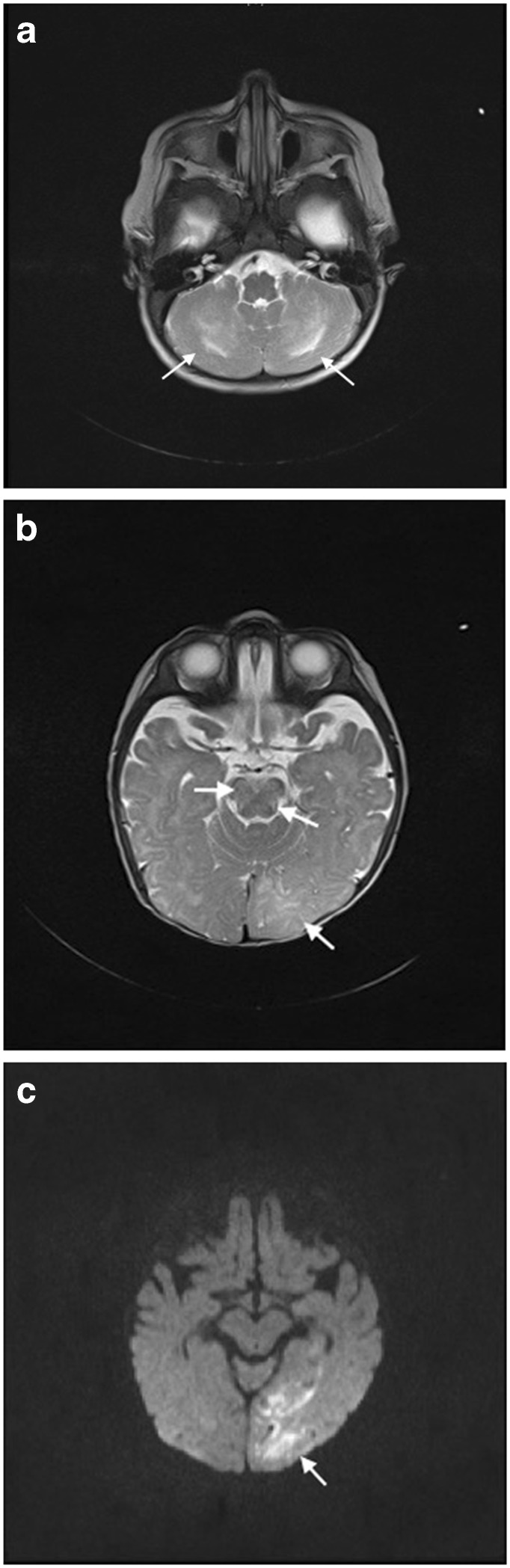
Neuroradiological MRI findings in HLH. **a** T2w image showing bilateral hyperintense lesions in the cerebellum. **b** T2w image with hyperintense signal and edema in the left posterior hemisphere and abnormalities in the brainstem. **c** Diffusion weighted imaging of the same region as in **b** with lesions imitating cerebral infarction.

MRI of the brain with gadolinium is the imaging modality of choice in situations in which CNS involvement in HLH is suspected (Figs. 1 and 2). Where MRI is not readily available, CT scans may provide valuable structural and other information, but cannot replace the detailed assessment available from MRI. The range of abnormalities seen on neuroimaging is broad. Descriptive information from retrospective case series including both primary and secondary forms of HLH suggests that multifocal and bilateral abnormalities seen on T2-weighted imaging are almost universally present in primary HLH (89%), with a high rate of symmetric involvement (53%), thus, distinguishing it from ADEM where symmetric involvement is infrequently seen [8]. Furthermore, large, ill-defined, confluent lesions are seen in up to 2/3 of an HLH population [8]. CNS hemorrhage was seen in 5/43 cases in one series of mixed primary and secondary HLH [10]. Chronic changes such as atrophy and calcifications are noted in several series, but information on timing of the imaging and relation to the use of agents such as steroids and in relation to onset of symptoms are not available in these reports [8–10, 38]. Administration of contrast is beneficial in this population, with one small case series suggesting the presence of nodular or ring (6/9), and/or leptomeningeal enhancement (5/9) in the majority of cases [38].

## Treatment of CNS HLH

### Review of the literature

The available literature offers no consensus on treatment directed at CNS involvement in HLH. Evidence-based guidelines are lacking and no clinical trials with a focus specifically on CNS disease have been conducted to date. In order to identify published cases of CNS HLH and its specific treatment, we searched the PubMed and Medline databases using the terms “Hemophagocytic lymphohistiocytosis,” “Familial hemophagocytic lymphohistiocytosis,” or “Macrophage activating syndrome.” The search included studies of both children and adults in the English literature and identified 15 papers focused on treatment of CNS involvement in HLH (summarized in Table 2). Most articles were retrospective case reports or case series.

**Table 2:** Studies describing treatment of CNS involvement in HLH

Reference	Study type	Study group	Treatment	Key results
Haddad et al. [9]	Case series Retrospective single center	29 (children)	Between 1981 and 1990, Etoposide and MTX IT; since 1991, CSA and ATG). During the maintenance therapy of both regimens, MTX IT was given usually every month. In nine cases, HSCT.	• The outcome of the 19 patients treated by systemic and intrathecal chemotherapy and/or immunosuppression exclusively was poor: all died following occurrence of multiple relapses or CNS disease progression in most cases. • 7/10 cases who had an HCST survived without neurological late effects. • In conclusion, HSCT is the best treatment of CNS disease progression and that it can result in cure when performed early enough after remission induction.
Ouachee-Chardin M et al. [39]	Case series Retrospective single center	48 (children) 38 with neurological disease before HSCT	HSCT	• HSCT, whatever the donor compatibility, is the treatment of choice for FHLH. It prevents relapses and CNS disease progression. • Only 2 (10%) of 21 survivors with neurologic HLH disease detected before HSCT had sequelae. In all others, a normal cognitive development has been observed with normal school performances within a follow-up now reaching 20 years
Mahlaoui et al. [17]	Case series Retrospective single center	38 (children) 19 with neurological disease	Combination of ATG with corticosteroids, CSA, and MTX IT	Treatment worked better for those without neurological disease: The probability of achieving CR for children with neurological disease was 58% (11 of 19) compared with 89% (17 of 19) in those without overt neurologic disease. In patients who did not receive a transplant shortly after ATG therapy, median duration of CR was 1.3 months with considerable variability (range 0.5–18 months).
Horne et al. [16]	Treatment study: HLH-94 Prospective multicenter	193 (children)	HLH-94 (combination of etoposide with corticosteroids, CSA, and MTX IT	Treatment worked best for those without neurologic symptoms and normal cerebrospinal fluid: The probability of being alive without neurological symptoms at two months after start of therapy was 66% (81/122) for those with either neurological symptoms and/or abnormal cerebrospinal fluid compared with 89% (63/71) in patients without any of these features.
Tateishi Y et al. [40]	Case series Retrospective single center	8 (children) 3 with CNS “failure”	Continuous hemodiafiltration using a polymethyl methacrylate membrane hemofilter (PMMA-CHDF) for cytokine removal in patients with refractory secondary HLH	Even in patients with severe HLH complicated with organ failure who are refractory to medical therapy, PMMA- CHDF may be effective in controlling HLH and contribute to survival: 2/3 with CNS “failure” went into remission. One died.
Sparber-Sauer et al. [41]	Case series Retrospective single center	18 (children) 3 with active CNS disease	HSCT	Patients with early relapse of primary HLH or with persistent CNS involvement may benefit from immediate HSCT. Both surviving patients with active CNS disease at HSCT are free of neurological sequelae.
Hu Y. et al. [42]	Case series Retrospective single center	15 (adults) 3 with neurological involvement	Cyclophosphamide, vincristine and prednisone combined chemotherapy (COP). Patients diagnosed with sHLH were enrolled and treated with the COP regimen as either initial or second-line therapy.	• COP chemotherapy had a relatively favourable effect for adult patients with sHLH, and the toxicities were tolerable • A CR was achieved in 7/15 patients (46.7%) • The 1-year overall survival was 66.7% • Conclusion: heterogeneous prognosis depends on the different aetiological factors for sHLH and choice of treatment
Rajajee et al. 2014 [43]	Case series Retrospective single center	40 (children) 7 with CNS disease	Secondary HLH treated in three different groups: 1. Children treated with IVIG of 1 gm/kg/day for 2 days, with dexamethasone at 10 mg/m^2^/day for 7 days followed by 6 mg/m^2^/day until complete clinical and lab response 2. Children treated with HLH-2004 protocol (etoposide, cyclosporine and dexamethasone) 3. Children treated with IVIG followed by HLH-2004 protocol	• Both IVIG therapy and HLH protocol 2004 were found to be equally efficient in the management of secondary HLH • In conclusion the use of IVIG therapy may be considered in all patients as the initial regimen and then followed by HLH 2004 if this fails
Marsh et al. [44]	Case series Retrospective multicenter	22 (children) Two with refractory CNS disease	Alemtuzumab	• This case series suggests that alemtuzumab therapy of refractory HLH results in improvement and survival to allogeneic HCT in most patients • There was a lack of adequate data to fully comment on the responsiveness of refractory CNS disease to alemtuzumab in these patients, other than the observation that no significant clinical improvement was noted on clinical examinations • One of the patients with refractory CNS disease died soon after alemtuzumab treatment
Deiva et al. [8]	Case series Retrospective single center	46 (children) 29 with neurological symptoms	Combination of ATG or alemtuzumab with corticosteroids, CSA, and MTX IT	21/29 children with neurological symptoms (72%) achieved complete or partial remission and received HSCT compared to 16/17 (94%) without neurological symptoms at onset.
Rachmandran et al. [6]	Case series Retrospective single center	43 (children) 12 with neurological symptoms	Steroids with or without IVIG were used commonly in the early phase of the disease. In total corticosteroids were used in 67%, IVIG in 64%. Cyclosporine was used in 33% and etoposide in 15%.	The overall mortality (24%) was lower than in other Asian case series. Better survival rate in this paper might be due to a high incidence of secondary HLH, early diagnosis and early institution of immunomodulatory treatment.
Koh et al. [22]	Case series Retrospective multicenter	251 (children) 81 with CNS disease	HLH-94- or HLA-2004-based immunochemotherapy	Treatment worked better for those without CNS disease: 5-year overall survival for those with CNS involvement was 56% compared to 76% for those without.
Dao et al. [23•]	Case series Prospective single center	89 (children) xx with CNS	• Suspected secondary HLH treated with either DX + CsA + etoposide or DX + CsA • No HSCT	The combination chemotherapy including etoposide was related to a favorable prognosis of survival in secondary HH patients: 40/50 (80%) vs 23/37 (62%)
Kim et al. [19]	Case series Retrospective single center	50 (children) 23 with CNS disease	During the study period, three different chemotherapy protocols were used: 1. Patients diagnosed November 1996–February 1997: immune therapy regimen combining treatment with steroids and ATG, followed by maintenance therapy with CSA and IT MTX 2. Patients diagnosed September 1995–November 2004: HLH 94 protocol 3. Patients diagnosed from March 2005 onwards: HLH 2004 protocol	Overall, patients with CNS disease achieve poorer outcomes than patients without CNS involvement.

GLOSSARY: *ATG* antithymocyte globulin, *CSA* cyclosporin A, *MTX IT* methotrexate intrathecal, *HSCT* hematopoietic stem cell transplantation

The majority of published reports describe the use of systemic steroids (primarily dexamethasone) combined with other immunosuppressive therapies (cyclosporine A and etoposide), as in the treatment protocols of HLH-94 and HLH-2004 [7, 15]. Both these protocols used intrathecal treatment weekly during the induction phase from weeks 3–6 in patients who had clinical symptoms of CNS disease progression after 2 weeks of systemic treatment, or in those with worsening or unchanged CSF pleocytosis. The rationale for not using intrathecal chemotherapy in all children with CNS involvement at therapy start was that the CNS symptoms improved with systemic therapy alone in most cases. The HLH-94 protocol [7] used intrathecal methotrexate alone while HLH-2004 [15] used intrathecal prednisolone in addition. Another option for HLH treatment is antithymocyte globulin (ATG rabbit) and methylprednisolone followed by cyclosporine A, which is given until hematopoietic stem cell transplant (HSCT), generally allowing tapering of methylprednisolone [17]. In this protocol, patients with CNS disease also receive intrathecal methotrexate and corticosteroids [17]. As both the definitions of CNS-HLH and the time points of treatment vary, direct comparison between these treatments is difficult. The HLH-94 protocol showed that 81/122 (66%) patients with any neurological involvement at onset of disease were alive and in complete remission at 2 months after start of treatment [7]. This is comparable to the ATG study [17] which stated that in patients with signs of overt neurologic disease the probability of achieving complete remission was 11/19 (58%) within a median time of 1.3 months.

Today, intrathecal methotrexate and corticosteroids (as described in HLH-2004 [15]) has become standard of care in the initial treatment of children with CNS-HLH. However, data on the value of intrathecal therapy in patients with CNS involvement is limited. In the first report on HLH-94, neurological alterations were reported in 35/109 (32%) of the patients at onset. In the 35 affected individuals, symptoms normalized in 21/31 (67%) survivors after 2 months of HLH-94 therapy. The rate of normalization was similar whether intrathecal therapy was used or not as an additional treatment to systemic corticosteroids, etoposide, and cyclosporin (10/15 versus 10/15, respectively) [45]. However, intrathecal methotrexate was not studied in a randomized fashion. Hence, additional studies will be required to better evaluate the value of intrathecal therapy in CNS-HLH.

Interestingly, a single report describes treatment with intrathecal rituximab in post-transplant lymphoproliferative disorder (PTLD) in the CNS, a lymphoproliferative disorder with similar features of CNS-HLH. PTLD is a very rare complication of HSCT. This study described successful treatment of CNS PTLD with intrathecal rituximab therapy in two children who had failed to respond to standard chemotherapy, intravenous rituximab and EBV specific cellular therapy [46]. For patients with HLH secondary to EBV-infection, rituximab has been shown to be beneficial for systemic symptoms [47, 48], but the literature does not provide specific results on CNS disease in these patients.

Alemtuzumab [49], a monoclonal anti-CD52 antibody, is another option that has been used for treatment of HLH [44]. This immunotherapy targets B and T lymphocytes and macrophages. A study on refractory HLH with alemtuzumab has yielded promising results [44], but there was a lack of adequate data to comment fully on the responsiveness of refractory CNS disease. Immunomodulating drugs such as IL-1 receptor antagonists have also been used to treat HLH/MAS with improvements described in case reports [50•, 51] and a case series with 12 patients [52]. However, data on CNS involvement were not available in any of these publications.

Two reports suggest that children with CNS HLH can be cured by initiation of HSCT soon after onset. Successful transplantation may prevent both reactivations and CNS disease progression [39] and may prevent the emergence of neurological late effects [41], though reactivation of CNS disease may occur after HSCT.

### Ongoing clinical trials

Hybrid immunotherapy for HLH (HIT-HLH, NCT01104025) and Euro-HIT, two clinical trials utilizing ATG, etoposide, and dexamethasone, recently closed and results are pending. Overall, the rationale for these trials was the potential additive or synergistic effects of combining anti-T cell serotherapy (ATG) with reduced dose-intensity etoposide in order to achieve sustained HLH suppression while minimizing myelosuppression. Because these trials utilize conventional agents with typical IT therapy in the case of CNS disease, it is not expected that they will provide new information regarding the treatment of CNS-HLH.

Another ongoing trial combines alemtuzumab, methylprednisone, and cyclosporine (NCT02472054). The rationale for this trial is based on the T cell depleting effects of alemtuzumab and prior reports of its activity in HLH [44, 53]. The trial is ongoing and results are not available.

Also ongoing is a trial (NCT01818492) testing a new anti-IFNγ monoclonal antibody NI-0501 for the treatment of HLH. This therapy represents a new and targeted approach for treating HLH based on preclinical data from animal models. Although the trial is ongoing and final results are pending, interim results were reported in abstract form (https://ash.confex.com/ash/2015/webprogram/Paper87376.html) in December 2015. This report revealed that three patients with CNS disease were treated and that two were possible to evaluate. Both had resolution of CNS signs and symptoms. At this time, it is not clear how useful NI-0501 will be for CNS-HLH, as results of the trial are still preliminary and these numbers are very small. Of note, a large drug such as an antibody would not ordinarily be expected to cross the blood-brain barrier. However, inflammation is known to open this barrier in other contexts and this also may occur in CNS-HLH.

Another very promising agent is the JAK1/2 inhibitor ruxolitinib shown in a recent study of two murine models of HLH to be effective. In the Rab27a-/- mice, CNS involvement was significantly reduced with ruxolitinib therapy [54]. To our knowledge, there is so far no clinical trial initiated with ruxolitinib.

### Treatment considerations—opinion statement

The first step towards optimal treatment of CNS-HLH is prompt and accurate diagnosis. This means looking for CNS involvement even when the patient does not present with obvious signs or symptoms. All patients should receive a brain MRI and lumbar puncture, including assessment of neuro-inflammatory markers if available, as soon as this can safely be performed after diagnosis of HLH. We regard presence of unequivocal neurological symptoms and/or signs, any abnormality in the CSF or brain MRI compatible with an inflammatory process to be consistent with a diagnosis of CNS-HLH. Therapy should be started in all HLH cases with neurological symptoms even if a lumbar puncture or MRI have not been obtained or results are still pending.

Although the optimal treatment for CNS-HLH is unknown, below, we present our approach to therapy, which is based on currently available evidence and/or personal experience. The algorithm in Fig. 1 represents the personal view of the authors how to approach the HLH patient with CNS disease. The treatment suggestions in Fig. 1 include HLH patients with genetic and acquired disease without a known underlying condition such as a malignancy, rheumatologic, or metabolic disease.

A steroid, preferably high-dose dexamethasone, is of importance in CNS-HLH treatment. Results of preclinical studies have shown that dexamethasone has a longer half-life in the CSF and better CSF penetration than does prednisone [55, 56], and in prospective randomized trials, dexamethasone yielded better control of CNS leukemia [57]. The highest standard dose of dexamethasone in the HLH-94/HLH-2004 protocols is 10 mg/m^2^ per day, but some authors have used 20 mg/m^2^ per day for limited time periods in patients with severe or refractory CNS-HLH.

Etoposide may be effective in the treatment of CNS-HLH. It has also recently been shown to be effective in a murine model of autoimmune encephalitis by selectively attacking activated T cells [58]. Treatment with etoposide at a reduced dosage (75–100 mg/m^2^) administered only once a week in combinations with corticosteroids can be used by in patients with MAS-HLH and CNS disease [59].

Our recommendation is that intrathecal Mtx and steroids (as described in HLH-2004) for CNS-HLH should be used as a first-line therapy. Treatment protocols are typically weekly for at least three doses and preferably until all CSF indices and CNS symptoms normalize. Surveillance CSF analyses should be obtained for 2–3 weeks afterwards and later if any symptoms reoccur. Brain MRI’s are typically abnormal for months after resolution of all other aspect of CNS disease so this should not be used in isolation for guiding subsequent therapy, unless clearly indicative of new or worsening problems. A recent case report described the use of intrathecal etoposide as successful salvage treatment for a patient with breast cancer and leptomeningeal metastasis [46]. One author also advocates intravenous thiotepa for refractory CNS-HLH since it readily crosses the blood brain barrier [60] and also leads to high drug levels in the CSF. [61, 62]. However, the use of intrathecal therapy for CNS-HLH is controversial. Because cells infiltrate the brain not only via the meninges but also via vessels, as seen in multiple sclerosis, some argue that invasive intrathecal therapy may not be needed. This is in line with the good response to systemic therapy alone in highly inflammatory CNS conditions other than HLH. There are also risks with intrathecal chemotherapy: neurological adverse effects are well described and may be expected [63]. There are also ongoing concerns regarding intrathecal exposure as a major contributor to CNS late effects in children [64]. However, intrathecal therapy may be necessary in patients in which treatment with dexamethasone, etoposide, or ATG offers good systemic control of HLH, but does not control CNS disease. Future studies are required to understand the value and risks of intrathecal therapy in CNS-HLH.

Even if a patient has responded well to initial therapy of HLH, reactivation of CNS-HLH by the time of HSCT is common. Early transplant in HLH can halt the progression of CNS disease [39, 41]. Therefore, even if HLH is still active, an early transplant should be considered as the risk of late effects is more severe than the risk of transplantation. In patients who have CNS involvement pre-transplant, surveillance LP’s after donor engraftment are advisable to monitor for recurrent/persistent CSF abnormalities. If CNS HLH recurs/worsens after HSCT, as indicated by clinical findings or CSF, additional IT and system therapy should be considered. Finally, all long-term survivors should have longitudinal follow-up for neurological late effects including cognitive and motor evaluations.

## Conclusion

CNS-HLH is a life-threatening condition, often but not always associated with systemic HLH, for which appropriate clinical, immunological, and radiological work-up is necessary. Current standard of therapy based on immuno-chemotherapy followed by HSCT in patients with primary disease leads to disease control in most, but not all patients. Novel therapeutic approaches are needed, some of which are currently being explored in ongoing clinical trials.
